# Is cannabidiol a drug acting on unconventional targets to control drug‐resistant epilepsy?

**DOI:** 10.1002/epi4.12376

**Published:** 2020-01-17

**Authors:** Luisa Rocha, Christian Lizette Frías‐Soria, José G. Ortiz, Jerónimo Auzmendi, Alberto Lazarowski

**Affiliations:** ^1^ Departamento de Farmacobiología Centro de Investigación y de Estudios Avanzados México City México; ^2^ Department of Pharmacology and Toxicology School of Medicine University of Puerto Rico San Juan Puerto Rico; ^3^ Departamento de Bioquímica Clínica Facultad de Farmacia y Bioquímica Instituto de Investigaciones en Fisiopatología y Bioquímica Clínica (INFIBIOC) Universidad de Buenos Aires Buenos Aires Argentina; ^4^ Consejo Nacional de Investigaciones Científicas y Técnicas (CONICET) Buenos Aires Argentina

**Keywords:** cannabidiol, cannabis, drug‐resistant epilepsy, P‐glycoprotein

## Abstract

Cannabis has been considered as a therapeutic strategy to control intractable epilepsy. Several cannabis components, especially cannabidiol (CBD), induce antiseizure effects. However, additional information is necessary to identify the types of epilepsies that can be controlled by these components and the mechanisms involved in these effects. This review presents a summary of the discussion carried out during the 2nd Latin American Workshop on Neurobiology of Epilepsy entitled “Cannabinoid and epilepsy: myths and realities.” This event was carried out during the 10th Latin American Epilepsy Congress in San José de Costa Rica (September 28, 2018). The review focuses to discuss the use of CBD as a new therapeutic strategy to control drug‐resistant epilepsy. It also indicates the necessity to consider the evaluation of unconventional targets such as P‐glycoprotein, to explain the effects of CBD in drug‐resistant epilepsy.

**Key points**

- Conflicting results exist about the use of artisanal cannabis to control drug‐resistant epilepsy
- Cannabidiol is a multitarget drug that represents a new hope to control drug‐resistant epilepsy
- Cannabidiol may act on unconventional central and peripheral targets to control drug‐resistant epilepsy

## 1 INTRODUCTION

Epilepsy is a neurological disease characterized by the presence of spontaneous and recurrent seizures.^1^ About 50 million people worldwide suffer epilepsy.^2^ Despite the development of new antiepileptic medications over the last decades, 30% of patients with epilepsy continue having seizures, resulting in the named drug‐resistant epilepsy. Drug‐resistant epilepsy is associated with comorbid psychiatric and psychological disorders, severe economic and social impairments, and high risk of suicide as well as sudden unexpected death (SUDEP).^3^, ^4^ The high prevalence of drug‐resistant epilepsy has generated the search of new solutions using old drugs.

## 2 IS CANNABIS AN OPTION FOR EPILEPSY?

Cannabis, one of the oldest plants that humans grow, was used in the Middle East to control nightly seizures around 1800 BC.^5^ Experimental evidence obtained during the 1970s‐1980s indicates that “phytocannabinoids” obtained from cannabis exerted anticonvulsant effects in experimental models of both, acute seizures^6^, ^7^, ^8^, ^9^ and epilepsy.^10^, ^11^, ^12^, ^13^, ^14^

During the last decade, the use of cannabis extracts has been of great interest in the control of drug‐resistant epilepsy, mainly in children with severe (catastrophic) epileptic syndromes, that is, neurological syndromes associated with seizures difficult to control and cognitive dysfunction such as Dravet (pathogenic variants in the sodium channel gene SCN1A) or Lennox‐Gastaut syndromes. Clinical evidence supports that pediatric and adult patients with refractory epileptic disorders may achieve a significant improvement with the administration of cannabis (Table 1). However, other studies indicate that the effectiveness of phytocannabinoids as antiseizure therapy is contradictory.^15^, ^16^ Based on the information described above, the National Academy of Science, Engineering and Medicine of the USA indicates that at present, the evidence to support the use of cannabinoids in epilepsy is insufficient.^17^

**Table 1:** Summary of the clinical studies evaluating the efficacy of cannabinoids in epilepsy and drug‐resistant seizures

Type of study	Type of epilepsy	Number of subjects, and age	Drugs	Doses	Treatment duration	Results	References
Combination of cannabinoids							
Open‐label, uncontrolled clinical trial	DRE, diverse etiology	n = 46 1‐20 y	CBD/THC (20:1) in enriched cannabis oil, oral administration	Tritiated administration starting with 2‐5 mg/kg/d sublingually and up to 50 mg/kg/d, plus THC (<1.35 mg/kg/d)	12 wk	≥50% reduction in the seizure frequency of 26 patients (56%)	127
Open‐label, uncontrolled clinical trial	Dravet syndrome with DRE	n = 19 1‐18 y	CBD/THC (50:1) in enriched cannabis oil, oral administration	Tritiated administration starting with 2‐5 mg/kg/d and up to 50 mg/kg/d, plus THC (0.27 mg/kg/d)	20 wk	≥50% reduction in the seizure frequency of 12 patients (63%)	128
Retrospective cohort study	DRE, diverse etiology	n = 74 1‐18 y	CBD/THC (20:1) in enriched cannabis oil, oral administration	Two CBD groups: 1‐10 mg/kg/d plus THC (<0.5 mg/kg/d) 10‐20 mg/kg/d plus THC (<0.5 mg/kg/d)	12‐48 wk	≥50% reduction in the seizure frequency in: 34 patients (46%) 4 patients (5%)	62
Retrospective cohort study	DRE, diverse etiology	n = 75 30 d‐18 y	Oral cannabis extracts: CBD alone CBD + other pCB THCA alone Other pCB	Variable, not specified	4‐96 wk	≥50% reduction in the seizure frequency of 25 patients (33%)	129
Case series	DRE of diverse etiology	n = 18 19‐50 y	Inhalated marijuana, smoked (n = 15) Oral marijuana (n = 2) Inhalated marijuana, vaporized (n = 1)	2.05 ± 1.87 g/d	4‐220 wk	Decrease of seizure frequency and severity	130
Cannabidiol							
Open‐label, expanded‐access study	Lennox‐Gastaut syndrome and Dravet syndrome	n = 152 1‐51 y	CBD oil, oral administration	Tritiated administration starting with 2‐10 mg/kg/d and up to 25 mg/kg/d	144 wk	≥50% reduction in the seizure frequency of 25 patients (49%)	131
Open‐label extension trial	Dravet syndrome	n = 264 2‐55 y	CBD oil, oral administration	Tritiated administration starting with 2.5 mg/kg/d and up to 30 mg/kg/d	48 wk	≥50% reduction in the seizure frequency of 41 patients (40%)	53
Open‐label, expanded‐access study	DRE, diverse etiology	n = 100 >1 y	CBD oil, oral administration	Tritiated administration starting with 5 mg/kg/d and up to 50 mg/kg/d	12‐48 wk	≥50% reduction in the seizure frequency of 57 patients (57%) Children responded to lower dosages	132
Prospective open‐label cohort study	DRE, diverse etiology	n = 40 <18 y	CBD oil, oral administration	Tritiated administration starting with 2‐5 mg/kg/d and up to 25 mg/kg/d	12 wk	Clinical improvement of 7 patients (17.5%, according to physician) or 12 patients (30%, according to caregivers)	133
Double‐blind, randomized placebo controlled trial	Lennox‐Gastaut syndrome	n = 225 2‐55 y	CBD oil, oral administration (n = 149) Placebo (n = 76)	Two CBD groups, tritiated administration starting with 2.5 mg/kg/d: And up to 10 mg/kg/d And up to 20 mg/kg/d	14 wk	≥50% reduction of seizure frequency of: 30 patients (39%) 26 patients (36%) And 11 patients (14%) of the placebo group	52
Open‐label, expanded‐access study	CDKL5 deficiency disorder and Aicardi syndrome, Dup15q syndrome, Doose syndrome	n = 46 1‐30 y	CBD oil, oral administration	Tritiated administration starting with 2‐5 mg/kg/d and up to 25 mg/kg/d	48 wk	≥50% reduction in the seizure frequency of 26 patients (57%)	61
Retrospective cohort study	DRE, diverse etiology	n = 108 <18 y	Artisanal CBD oil Artisanal CBD oil + Clobazam Clobazam alone	CBD average dose of 2.9 mg/kg/d CBD average dose of 5.8 mg/kg/d Clobazam average dose of 1.5 ± 1.4 mg/kg/d	52.8 wk 64 wk 120 wk	≥50% reduction in seizure frequency of: 16 patients (33%) 24 patients (44%) 28 patients (38%)	134
Open‐label, uncontrolled clinical trial	DRE, diverse etiology	n = 26 1‐17 y	CBD oil, oral administration	Tritiated administration starting with 5 mg/kg/d and up to 25 mg/kg/d	16‐212 wk	≥50% reduction in the seizure frequency of 7 patients (26.9%) at the end of the study	135
Open‐label, uncontrolled clinical trial	DRE, diverse etiology	n = 132 >1 y	CBD oil, oral administration	Tritiated administration starting with 5 mg/kg/d and up to 50 mg/kg/d	12‐48 wk	About 50% of the participants achieved ≥50% reduction in seizure frequency	136
Double‐blind, randomized placebo controlled trial	Lennox‐Gastaut syndrome	n = 171 2‐55 y	CBD oil, oral administration (n = 86) Placebo (n = 85)	Tritiated administration starting with 2‐5 mg/kg/d and up to 20 mg/kg/d	14 wk	≥50% reduction in the seizure frequency of: CBD, 38 patients (44%) Placebo, 20 patients (24%)	52
Double‐blind, randomized placebo controlled trial	Dravet syndrome	n = 120 2‐18 y	CBD oil, oral administration (n = 61) Placebo (n = 59)	20 mg/kg/d	14 wk	≥50% reduction in seizure frequency of: CBD, 22 patients (43%) Placebo, 15 patients (27%)	137
Case series	Febrile Infection‐Related Epilepsy Syndrome (FIRES)	n = 5 Children, age not specified	CBD oil, oral administration	15‐20 mg/kg/d	48 wk	Reduction in seizure frequency and severity	138
Case series	Refractory seizures in Sturge‐Weber syndrome	n = 5 1 mo‐45 y	CBD oil, oral administration	Tritiated administration starting with 5 mg/kg/d and up to 25 mg/kg/d	14‐80 wk	≥50% reduction in seizure frequency of 3 patients (60%) with bilateral brain involvement	139
Open‐label, uncontrolled clinical trial	Epilepsy of diverse etiology	n = 48 1‐30 y	CBD oil, oral administration	Tritiated administration starting with 2‐5 mg/kg/d and up to 50 mg/kg/d	4 wk	≥50% reduction in seizure frequency of 20 patients (41.7%), with improvement in memory and other cognitive functions	140
Case series	Brain tumor‐related epilepsy	n = 3 17‐40 y	CBD oil, oral administration	Tritiated administration starting with 5 mg/kg/d and up to 50 mg/kg/d	8‐44 wk	Reduction in seizure frequency and severity of 2 patients	141
Double‐blind, randomized placebo controlled trial	Focal seizures, DRE	n = 186 18‐71 y	Transdermal gel (CBD 4.2%), local administration Placebo	Two CBD groups: 195 mg every 12 h 97.5 mg every 12 h	12 wk	CBD and placebo showed similar effect	142
Open‐label, uncontrolled clinical trial	DRE, diverse etiology	n = 137 1‐30 y	CBD oil, oral administration	Tritiated administration starting with 2‐5 mg/kg/d and up to 25‐50 mg/kg/d	12 wk	≥50% reduction in seizure frequency of 51 patients (37%)	143
Open‐label, expanded‐access study	TSC and DRE	n = 18 2‐31 y	CBD oil, oral administration	Tritiated administration starting with 5 mg/kg/d and up to 25‐50 mg/kg/d	24‐48 wk	≥50% reduction in seizure frequency of 4 patients (50%) at the end of the study	144
Double‐blind, randomized placebo controlled trial	Temporal lobe epilepsy with secondarily generalized seizures	n = 15 14‐49 y	CBD, capsules for oral administration (n = 8) Placebo (n = 8)	200‐300 mg/d	8‐18 wk	Clinical improvement in: CBD, 4 patients (50%) Placebo, 1 patient (12%)	45, 145
Cannabidivarin							
Double‐blind, randomized placebo controlled trial	Focal epilepsy, DRE	n = 32 18‐65 y	CBDV (GWP42006), oral administration Placebo	800 mg/d	2 wk	Results not published	146
Double‐blind, randomized placebo controlled trial	Focal epilepsy, DRE	n = 162 18‐65 y	CBDV (GWP42006), oral administration Placebo	Tritiated administration starting with 800 and up to 1600 mg/d	8 wk	Seizure frequency decrease 40% in both groups	147
Tetrahydrocannabidiol							
Case series	Epilepsy and MR	n = 5 Children, age not specified	THC isomers, route of administration not described	Up to 4 mg/d	3‐7 wk	Clinical improvement in 2 patients (40%)	148
Case series	Epilepsy and MR	n = 6 8‐14 y	THC oil, oral administration	Up to 0.12 mg/kg/d	Not specified	Clinical improvement in 4 patients (67%)	149

Abbreviations: AEDs, Antiepileptic drugs; artisanal CBD oil, ten different CBD products; CBD, cannabidiol; CBDV, cannabidivarin; DRE, drug‐resistant epilepsy; mo, months; MR, mental retardation; OCE, oral cannabis extracts; pCB, phytocannabinoid; SD, standard deviation; THC, Δ9‐tetrahydrocannabidiol; THCA, tetrahydrocannabinolic acid; TSC, tuberous sclerosis complex; wk, weeks; y, years.

The conflicting results about cannabis failure or success in epilepsy can be explained by several circumstances. First of all, the pharmacokinetics and pharmacodynamics of cannabinoids depend on the formulation and route of administration. Indeed, some effects of cannabinoids are explained by pharmacokinetic interactions such as inhibition or induction of enzymes involved in drug metabolism.^18^ Other important issue is that the bioavailability of cannabinoids applied by oral administration in liquid formulations augments with the fed state, especially with high‐fat meals.^19^

On the other hand, the effects of cannabis on specific types of epilepsy are unknown. Concerning this issue, few studies exist about the effects of cannabis extracts in temporal lobe epilepsy, the most common drug‐resistant epileptic syndrome in adults (see below). The influence of clinical factors (age of patient, gender, etc) in the efficacy of cannabis has not been considered in clinical studies. Other factor that can modify the effects of cannabis is its coadministration with other drugs, especially antiseizure drugs. Few data exist about the pharmacokinetic and pharmacodynamic interaction between cannabis and other drugs.^20^ However, no information exists about the pharmacokinetic and pharmacodynamic interaction between cannabis and antiseizure drugs.

An important condition that explains the contradictory effects of cannabis on epilepsy is that this plant contains more than 480 compounds, including noncannabinoids such as prenylated flavonoids, stilbenoids derivatives, and lignanammides.^21^ The content of the different chemical components in cannabis depends on each species. *Cannabis ruderalis* contains the lowest concentrations of Δ9‐tetrahydrocannabidiol (THC).^22^ European *Cannabis sativa* contains more cannabidiol (CBD) than THC, whereas Asian *Cannabis indica* has more THC than CBD.^23^

Although artisanal cannabis is considered a “miracle therapy,” at present there are not regulations to maintain the quality and purity of the drug during the obtaining procedure. Artisanal cannabis oil may contain abiotic (dust, fertilizers) and biotic (ie, insect, fungi, bacteria) contaminants, heavy metals, pesticides, etc,^24^ a situation that represents a high risk to the health of patients. Unfortunately, the evaluation of the effects of artisanal cannabis is difficult, has yielded controversial results, and lacks controlled clinical studies.^25^

There is an apparent disregard for long‐term use of cannabis. Long‐term cannabis administration augments the risk of addiction and is associated with side effects such as chronic bronchitis. It also enhances the possibility to present psychosis and schizophrenia in persons with a predisposition to such disorders. Adolescents are more vulnerable to the side effects of chronic cannabis use as there is altered brain development, cognitive impairment, poor academic outcomes, etc^26^. According to this information, it is evident the necessity to obtain more information concerning the beneficial effects of cannabis oil in the control of drug‐resistant epilepsy and stablish standardized procedures to obtain homogeneous products. In addition, it is essential to elucidate the contribution of each compound in the therapeutic effects induced by cannabis.

## 3 IS CANNABIDIOL A NEW HOPE FOR DRUG‐RESISTANT EPILEPSY?

At present, there are studies indicating that some cannabis products may induce antiepileptic effects. These products are THC, CBD, Δ9‐tetrahydrocannabivarin, cannabidivarin, and Δ9‐tetrahydrocannabinolic acid.^27^, ^28^ The main phytocannabinoids evaluated with this purpose are THC and CBD. THC is an active ingredient of cannabis plant that induces psychoactive effects, augments oxidative stress, and produces mitochondrial dysfunction in the brain, conditions that increase the risk to stroke and brain damage.^29^, ^30^ For these reasons, low interest exists about THC as an antiseizure drug.

Cannabidiol is the most abundant phytocannabinoid in cannabis. It has a terpenophenolic structure and hydroxyl groups in carbons 1 and 3.^31^ CBD shares lipophilic characteristics with the rest of the cannabinoids, lacks psychoactive effects,^32^ and induces neuroprotective effects.^33^

**Figure 1:**
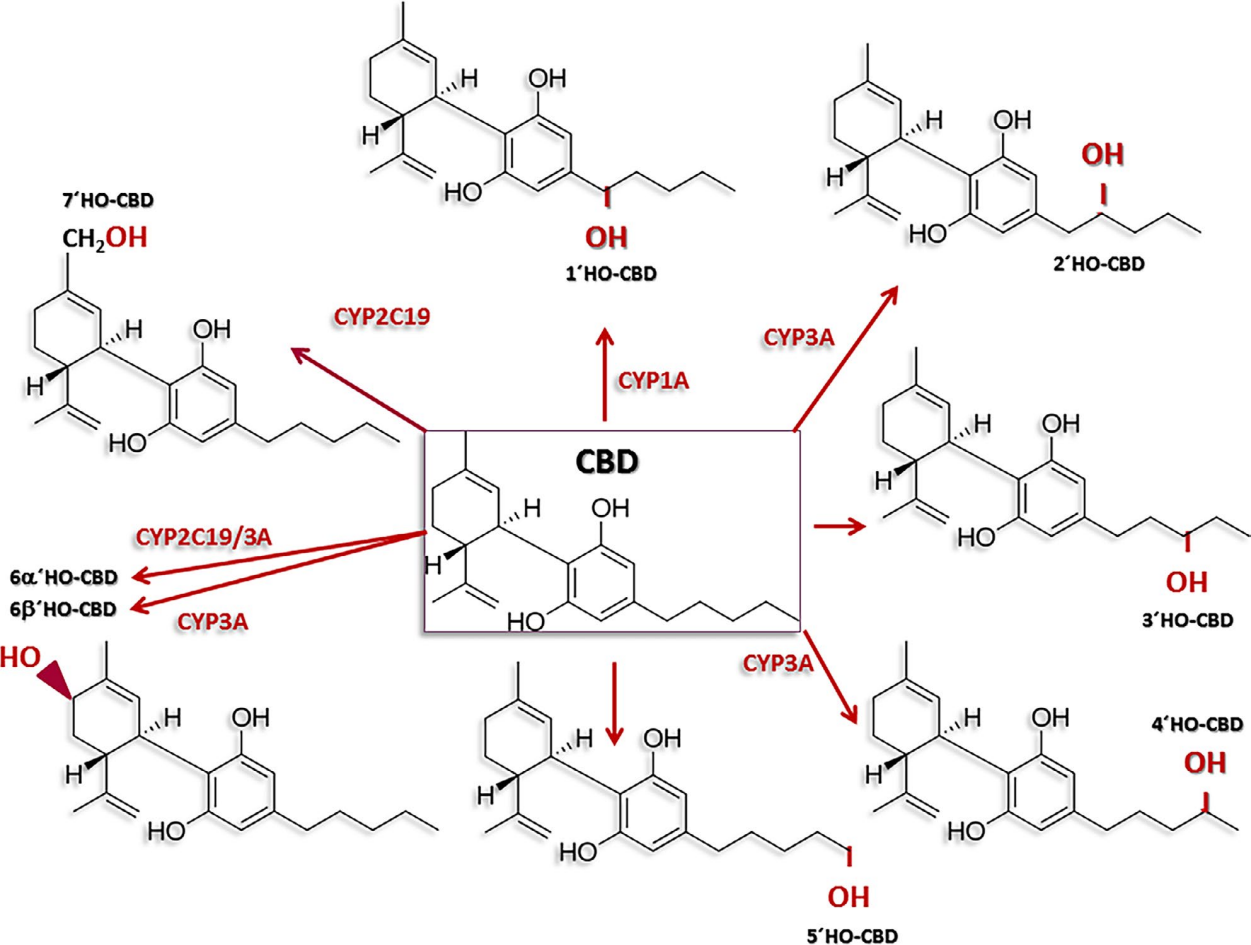
Schematic diagram indicating the different enzymes of the cytochrome P450 family (CYP450) involved in the metabolism of cannabidiol (CBD)

The metabolism of CBD comprises oxidation and hydroxylation through different enzymes of the cytochrome P450 family (CYP450) (Figure 1).^34^, ^35^, ^36^ In vitro studies using human liver microsomes revealed that 6α‐hydroxylation of CBD is mediated by CYP3A4 and CYP2C19 isoforms, 6β‐hydroxylation is induced by CYP3A4, whereas 7‐hydroxylation is mediated by CYP2C19.^35^ Glucuronosyltransferase and sulfotransferases enzymes are also involved in the metabolism of CBD.^36^, ^37^ At present, more than fifty metabolites from CBD have been identified in urine.^34^, ^38^ The most abundant metabolites are 7‐carboxy‐cannabidiol (7‐COOH‐CBD), 7‐hydroxy‐cannabidiol (7‐OH‐CBD), and 6‐hydroxy‐cannabidiol (6‐OH‐CBD). Although the biological activity of many of CBD metabolites is unknown,^36^, ^39^ preclinical studies suggest that 7‐OH‐CBD obtained from humans induces anticonvulsant effects in mice.^40^

On the other hand, CBD is a potent inhibitor of CYP1A1, CYP2B6, CYP2D6, and CYP2C19 with a subsequent reduction in the metabolism of some drugs. This condition explains the increases in the serum levels of THC, topiramate, rufinamide, clobazam, and N‐desmethylclobazam when they are coadministered with CBD.^41^, ^42^, ^43^ This effect is more evident when the drugs are oral administered.^44^ These studies lead to suggest that CBD augments the effects of antiseizure drugs. This idea is supported by the early observational clinical study carried out by Cunha et al,^45^ who described for the first time that the chronic administration of CBD reduced the seizure activity in seven of eight patients with drug‐resistant temporal lobe epilepsy. During the CBD treatment, the patients received the administration of the antiseizure drugs prescribed before the study. According to this information, it is evident the necessity of clinical studies focused to determine the effects of CBD in different experimental models of drug‐resistant epilepsy and its pharmacokinetic interactions with other drugs.

Several studies support that CBD could be effective in the control of epilepsy.^46^ Results obtained from experimental models reveal that CBD reduces the seizure activity^47^, ^48^, ^49^ and delays the epileptogenesis process, effects associated with neuroprotection.^50^, ^51^ CBD in oral solution (Epidiolex^®^) is considered a therapy to control seizures associated with the Lennox‐Gastaut syndrome,^52^ Dravet syndrome,^53^ and infantile spasms.^54^ Indeed, the US Food and Drug Administration (FDA) recently approved Epidiolex for the control of seizures associated with Lennox‐Gastaut syndrome and Dravet syndrome, in children (2 years of age and older) and adults.^55^

The antiepileptic, anxiolytic, antipsychotic, and neuroprotective effects induced by CBD lead to suggest that it is an excellent candidate to control drug‐resistant epilepsy and comorbid disorders.^56^ This notion is supported by results obtained from experimental models of temporal lobe epilepsy, a neurological disorder with a high prevalence of drug resistance and comorbid psychiatric symptoms.^57^ CBD induces neuroprotection, decreased neuronal excitability, and avoids cell death in the hippocampus of animals with temporal lobe epilepsy.^50^, ^58^ However, the effects of CBD in other types of drug‐resistant epilepsy are not conclusive due to the presence of subjects who do not respond to the treatment.^59^, ^60^, ^61^, ^62^

Clinical data in humans indicate that CBD‐rich extracts are more effective to reduce the seizure frequency when compared with purified CBD. In addition, CBD‐rich extracts reduce the seizure activity with a significantly lower average daily dose, supporting a higher potency when compared with purified CBD. These effects are associated with side effects such as appetite alterations, nausea, diarrhea and other gastrointestinal alterations, sleepiness, weight changes, and fatigue, among others.^63^ It is known that THC augments the analgesic effects of CBD.^64^ On the other hand, CBD potentiates or reduces some effects induced by THC.^65^ However, it is unknown whether the THC and CBD interaction facilitates or reduces the seizure activity.

## 4 CONVENTIONAL EFFECTS THAT EXPLAIN THE ANTIEPILEPTIC EFFECTS OF CANNABIDIOL

**Figure 2:**
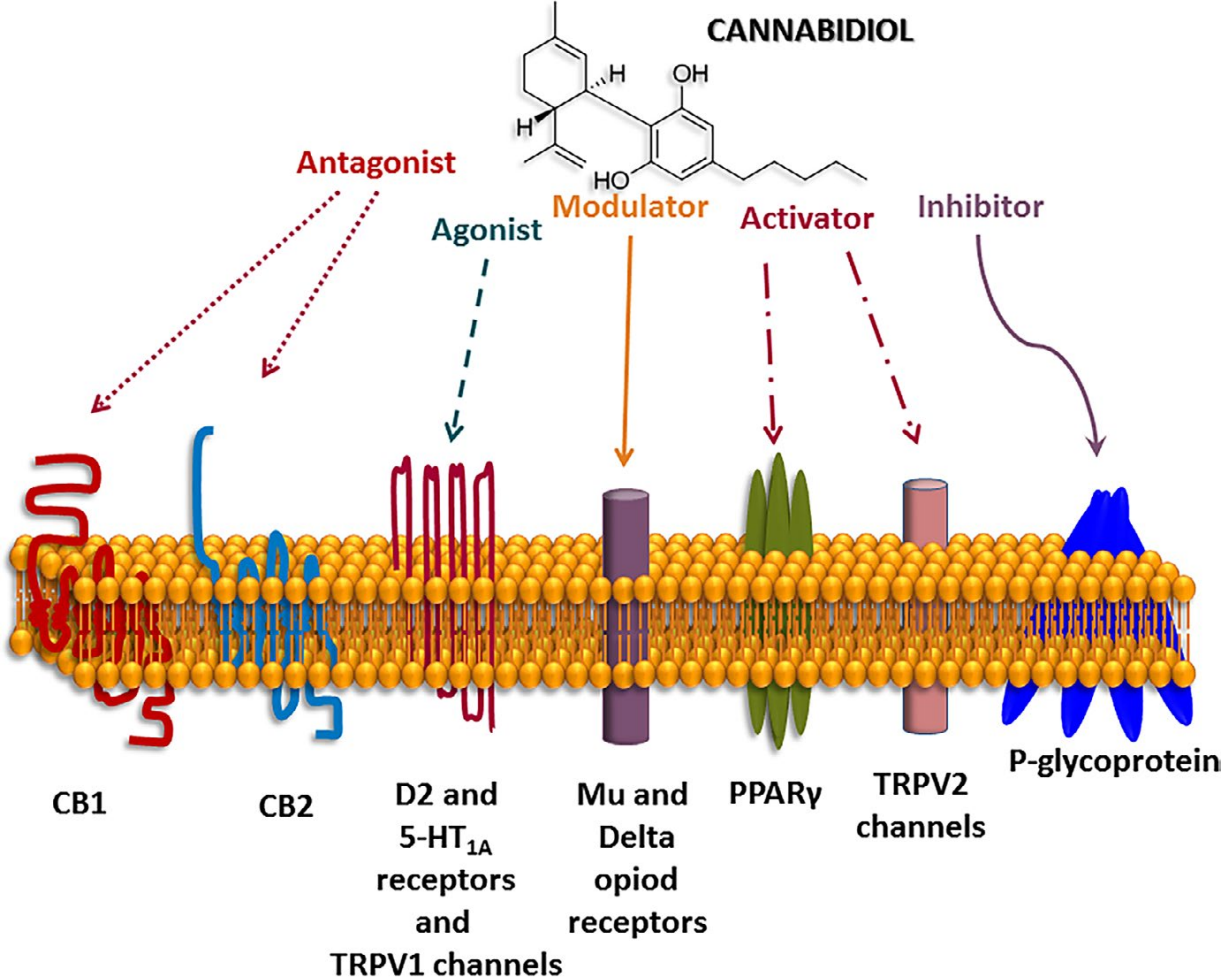
Mechanisms of action of cannabidiol (CBD) on different receptors, channels, and P‐glycoprotein transporter

Different targets are involved in the mechanisms by which CBD induces antiepileptic effects. Computational analysis and ligand displacement assays revealed that CBD augments the levels of endocannabinoids (anandamide) as result of the inhibition of the fatty acid‐binding proteins (FABPs) that mediate the anandamide transport to its catabolite enzyme (fatty acid amide hydrolase [FAAH]).^43^ On the other hand, experiments indicate that CBD is a very low‐affinity ligand at CB1 and CB2 receptors inducing antagonism.^66^ It acts as agonist on D2 (partial agonist)^67^ and 5‐HT_1A_ receptors.^68^, ^69^ CBD is an agonist of TRPV1 channels^27^ and activator of TRPV2 channels.^70^ It is an allosteric modulator of mu‐ and delta‐opioid receptors.^71^ CBD induces resting‐state blockage of sodium channels and blocks the voltage‐gated potassium channel subunit Kv2.1.^72^ Studies indicate that CBD produces neuroprotection by activation of CB2 and adenosine A2 receptors.^73^ In experimental models of pain, CBD induces analgesic effects through the activation of 5HT_1A_ and TRPV1 channels.^74^, ^75^ Using blood‐brain barrier (BBB) modeled with human brain microvascular endothelial cell and astrocyte co‐cultures, it was found that CBD induces neuroprotection in ischemic stroke by activation of PPARγ and 5‐HT_1A_ receptors.^76^ CBD injected into the dorsolateral periaqueductal gray of rats exerts anxiolytic‐like effects through the activation of 5‐HT_1A_ receptors (Figure 2).^77^

CBD has been suggested to play a critical role in the glutamatergic neurotransmission. However, the findings are controversial. CBD induces antidepressant‐like effects associated with enhanced serotonin and glutamate neurotransmission in a mouse model of depression.^78^ Other studies indicate that CBD reduces the overactivity of NMDA receptors through the antagonism of the sigma 1 receptors (σ1R).^79^ CBD also reduces glutamate release and protects from convulsive activity in an experimental model of seizures induced by cocaine.^80^ The effect of CBD on the glutamatergic neurotransmission is relevant because excess of extracellular levels of glutamate is associated with recurrent seizures and chronic epilepsy.^81^ Indeed, the blockage of NMDA receptors can prevent and in some cases reverse certain pathological conditions associated with neurological disorders, including epilepsy.^82^, ^83^

High glutamatergic neurotransmission, neuroinflammation, and oxidative stress are interconnected phenomena that occur in the brain of subjects with epilepsy. Interestingly, the increase in the oxidative stress and neuroinflammation associated with drug‐resistant epilepsy can be reverted when the seizure activity decreases as result of the surgical resection of the epileptic foci.^84^, ^85^, ^86^

Seizure‐induced neuroinflammation is a condition associated with the increase of cytokines such as interleukin (IL)‐1β, tumor necrosis factor (TNF), transforming growth factor (TGF)‐β, and danger signals such as High Mobility Group Box 1 (HMGB1). The activation of cytokines may underlie hyperexcitability and neurotoxicity, a situation that facilitates the epileptic activity.^87^, ^88^, ^89^ Concerning this issue, it is described that IL‐1β induced in activated astrocytes and microglia contributes to the occurrence of seizure activity.^90^, ^91^ IL‐1β and TNF produce excitatory effects by enhancement of Ca^2+^ influx and extracellular levels of glutamate with a subsequent production of hydroxyl radicals. TNF also modifies the glutamate subunit receptor composition of neurons and augments the glutamate release from microglia. In astrocytes, TNF enhances Ca^2+^ mobilization with a subsequent cyclooxygenase enzyme‐2 (COX‐2) activation, prostaglandin‐2 synthesis, and glutamate release.^92^, ^93^

Oxidative stress is a condition detected during epileptogenesis and chronic epilepsy. It is a consequence of mitochondrial dysfunction and increased activity of nicotinamide adenine dinucleotide phosphate oxidase (NOX), xanthine oxidase, and inducible nitric oxide synthase (iNOS) that result in the production of reactive oxygen species (ROS) and reactive nitrogen species (RNS). Oxidant stress facilitates inflammation through the induction of COX‐2 gene expression and ictogenic cytokines.^93^, ^94^ The high glutamate release and NMDA receptor activation in chronic epilepsy can also facilitate oxidative mechanisms and neurotoxicity.^95^, ^96^

Experimental evidence supports that CBD represents a novel strategy to reduce oxidative stress, excitotoxicity, and neuroinflammation in neurodegenerative disorders. CBD decreases oxidative stress, mitochondrial dysfunction and reactive oxygen species generation, effects associated with reduced neuroinflammation.^97^ CBD also augments microglial phagocytosis by the modification of TRPV channel activity.^98^ It induces anti‐inflammatory effects by decreasing the plasma levels of prostaglandin E2, the production of free radicals, and the activity of COX‐1/COX‐2.^99^, ^100^ CBD reduces neuronal damage, astrogliosis, excitotoxicity, and neuroinflammation in experimental models of ischemia.^101^ However, the immune effect of CBD can vary depending on the concentrations administered as well as the type and/or magnitude of stimulus.^102^

## 5 CANNABIDIOL MAY ACT ON UNCONVENTIONAL CENTRAL AND PERIPHERAL TARGETS TO CONTROL DRUG‐RESISTANT EPILEPSY

P‐glycoprotein is a BBB efflux transporter that limits drug accumulation in the brain. Its overexpression at the luminal side of the BBB is associated with drug‐resistant epilepsy because it results in a low penetration of antiseizure drugs into the brain.^103^, ^104^ P‐glycoprotein is also overexpressed in astrocytes and neurons in brain tissue obtained from patients and animals with drug‐resistant epilepsy.^105^, ^106^

The enhanced extracellular levels of glutamate produced in the brain of subjects with drug‐resistant epilepsy^107^ represent a mechanism that facilitates the overexpression of P‐glycoprotein in a COX‐2‐dependent manner.^108^ P‐glycoprotein overexpression in cells of the BBB can also result from chronic oxidative stress or prolonged neuroinflammation.^109^, ^110^ According to several experimental evidence, the administration of inhibitors of P‐glycoprotein function or expression represents a potential therapeutic strategy to control drug‐resistant epilepsy.^81^, ^111^ Concerning this issue, the use of celecoxib, a specific COX‐2 inhibitor, reverts the P‐glycoprotein overexpression and facilitates brain delivery of antiseizure drugs in animals with epilepsy.^112^ The administration of P‐glycoprotein inhibitors such as verapamil induces encouraging effects in patients with drug‐resistant epilepsy. However, these drugs may induce significant side effects that restrict their clinical application.^113^ Experimental evidence also supports that a better control of drug‐resistant epilepsy could be obtained if antiseizure drugs are associated with P‐glycoprotein inhibitors.^111^, ^114^, ^115^

Studies indicate that CBD down‐regulates the protein and mRNA expression of P‐glycoprotein and inhibits its efflux function in trophoblast cell lines.^116^ CBD also interacts with a specific site of the P‐glycoprotein interfering with the ATPase activity stimulated by substrates and consequently decreasing the energy required for their transport in Caco‐2 and LLC‐PK1/MDR1 cells.^117^ The inhibitory effect of CBD on P‐glycoprotein is evident after prolonged, but not short‐term exposure, in cells CEM/VLB_100_ that overexpress this transporter.^118^

It is important to mention that CBD is not a substrate of P‐glycoprotein. In mice, experiments revealed that the overexpression of this transporter at BBB does not limit the brain uptake of CBD.^119^ This condition associated with the inhibitory effect of P‐glycoprotein at BBB plus its anticonvulsant and neuroprotective effects suggests that CBD can be an attractive adjunctive therapy to control drug‐resistant epilepsy. However, further studies are necessary to demonstrate that the exposure to CBD blocks the activity of P‐glycoprotein and/or reverts its overexpression in neurons and astrocytes in brain tissue obtained from subjects with drug‐resistant epilepsy.

The overexpression of P‐glycoprotein in neurons is associated with high membrane depolarization, a condition that may facilitate the epileptiform activity.^120^ Interestingly, P‐glycoprotein overexpression is also induced in cardiomyocytes of subjects submitted to repetitive convulsive seizures. This condition is related with electrocardiographic (ECG) alterations and SUDEP as consequence of a depolarizing role in cardiomyocytes.^121^ If CBD is able to revert the P‐glycoprotein overexpression and its depolarizing condition in heart, it may represent a novel strategy to reduce SUDEP in drug‐resistant epilepsy. However, further experiments are necessary to support this hypothesis.

In addition to the pharmacological mechanisms previously described, CBD also induces epigenetic changes associated with neuroprotective effects. Concerning this issue, it is known that iron accumulation in brain regions is a condition that contributes to neurodegeneration. CBD restores the basal levels of hippocampal dynamin‐1‐like protein (DNM1L), caspase 3, and synaptophysin in animals with cell damage subsequent to iron loading.^122^ These effects are also associated with reversion of the iron‐induced mitochondrial deoxyribonucleic acid (mtDNA) deletions, the decreased epigenetic modulation of mtDNA, as well as restauration of the mitochondrial ferritin levels and succinate dehydrogenase activity.^123^ According to these studies, CBD induces epigenetic effects that results in the restauration of the normal cellular function and neuroprotection. Epigenetic effects induced by CBD hold promise as a future therapeutic strategy for drug‐resistant epilepsy. However, further research is essential to determine side effects induced by CBD when applied chronically, alone or combined with other antiseizure drugs inducing epigenetic effects such as valproic acid.^124^

On the other hand, the repeated coadministration of CBD with THC induces histone 3 acetylation (H3K9/14ac) in the ventral tegmental area, an epigenetic effect related with addiction processes.^125^ These effects should be considered when apply CBD chronically because H3K9 acetylation is significantly augmented as result of seizure activity, a condition associated with the activation of TLR4 and subsequent inflammation process.^126^

## 6 CONCLUSIONS

In the modern era of highly effective and specific therapies targeted to treat different disorders, efforts have to focus to identify the real composition of the used extracts of phytocannabinoids. Concerning CBD, it is evident that it induces therapeutic effects that can be applied to control drug‐resistant epilepsy. However, it is necessary to identify the types of epilepsy responsive to the beneficial effects of CBD. In addition, it is essential to know its pharmacokinetic, side effects as well as the cellular and molecular effects induced by its repetitive and long‐term administration, alone and associated with antiseizure drugs.

Other important issue to consider is the evaluation of unconventional targets and mechanisms of action of CBD and other cannabinoids to control drug‐resistant epilepsy and reduce fatal complications such as SUDEP. Several studies support that CBD decreases the expression and function of P‐glycoprotein in different cell types. If CBD is able to diminish the overexpression of this transporter in the brain of patients with drug‐resistant epilepsy, it could be used as adjunctive therapy to better biodistribution and CNS access of antiseizure drugs.

Finally, a scientific validation of the antiepileptic properties of the different cannabinoids and their metabolites as well as terpenes (alone and in combination) would benefit in the control of different types of epilepsy. This validation should consider the genetic background to understand the patients’ response to cannabinoids.

## CONFLICT OF INTEREST

None of the authors has any conflict of interest to disclose. The authors confirm that they have read the Journal's position on issues involved in ethical publication and affirm that this report is consistent with those guidelines. The present study is consistent with the Journal's guidelines for ethical publication.
